# Dorsum Sellae Morphology in Chiari Malformation Type I: A CT-Based Study

**DOI:** 10.3390/diagnostics16121765

**Published:** 2026-06-08

**Authors:** Burak Bahadır, Nur Hürsoy, Fatma Betül Saylak, Mert Yiğit, Ece Uzer, Pelin Ayanoğlu Gelişkan, Eda Aslanbaba Bahadır, Elif Banu Güler Oklaz, Tunahan Aksakallı, Ömer Faruk Türkoğlu, Orhan Beger

**Affiliations:** 1 Department of Neurosurgery, Ankara Bilkent City Research and Training Hospital, University of Health Sciences, Ankara 06800, Türkiye; 2 Department of Radiology, Faculty of Medicine, Recep Tayyip Erdoğan University, Rize 53100, Türkiye; 3 Department of Neurosurgery, Çankırı State Hospital, Çankırı 18100, Türkiye; 4 Department of Neurosurgery, Ankara Sincan Research and Training Hospital, Ankara 06949, Türkiye; 5 Department of Neurology, Ankara Golbasi Şehit Ahmet Özsoy State Hospital, Ankara 06830, Türkiye; edaaslanbaba@gmail.com; 6 Department of Radiology, Ağrı Doğubeyazıt Dr Yaşar Eryılmaz State Hospital, Ağrı 04402, Türkiye; 7 Department of Anatomy, Faculty of Medicine, Gaziantep University, Gaziantep 27310, Türkiye

**Keywords:** Chiari malformation type I, dorsum sellae, computed tomography, skull base, morphometry, pneumatization

## Abstract

**Objective**: To evaluate the morphology of the dorsum sellae (DS) on computed tomography (CT) in patients with Chiari malformation type I (C1M) and to compare the findings with those of controls. **Methods**: This retrospective case–control study included 120 individuals who underwent cranial CT, comprising 60 patients with C1M and 60 control subjects. **Results**: Several morphometric features of the DS differed significantly between the C1M and control groups. In the C1M group, the distance between DS and foramen magnum was shorter (43.15 vs. 46.00 mm, *p* < 0.001), whereas the distance between DS and anterior clinoid process (17.20 vs. 15.10 mm, *p* < 0.001) and the angle between the crista galli, DS, and foramen magnum (133.44° vs. 125.30°, *p* < 0.001) were greater. In addition, the transverse width of the DS was smaller, whereas midline height, bilateral lateral heights, and anteroposterior thickness were greater in the C1M group (all *p* < 0.001). DS pneumatization was also more frequent in patients with C1M (23.3% vs. 5.0%, *p* = 0.007). **Conclusions**: Distinct DS morphometric characteristics and an increased frequency of DS pneumatization were observed in patients with C1M. These findings may contribute to a better understanding of skull base anatomy in C1M.

## 1. Introduction

The dorsum sellae (DS) is a centrally positioned osseous structure of the cranial base that forms the posterior boundary of the sella turcica [1,2]. At its superolateral margins, it gives rise to the posterior clinoid processes (PCPs), which are important anatomical landmarks in the parasellar region [2]. Reported anatomical variations of the DS are relatively uncommon and mainly include agenesis and pneumatization [1,3]. Detailed knowledge of DS morphology is particularly important for neurosurgeons performing skull base procedures, including posterior clinoidectomy, as this region may be involved in the surgical management of lesions such as giant basilar tip aneurysms. In addition to facilitating surgical exposure, precise anatomical understanding may help reduce the risk of injury to adjacent critical neurovascular structures, such as the basilar artery [4,5,6,7,8]. Despite its clinical relevance, the available literature provides limited morphological data on the DS [9,10]. Therefore, further characterization of DS anatomy, particularly in specific populations such as patients with Chiari malformation Type I (C1M), may contribute to a more accurate understanding of its morphological features and improve its clinical interpretation.

Available evidence suggests that C1M may influence the osseous architecture of the entire cranial base [11,12]. In a pioneering study, Nwotchouang et al. [11] compared sphenoid sinus morphology between patients with C1M and controls and reported a 38% greater sinus volume in the C1M group, implying that adjacent structures surrounding the sinus, including the sella turcica, may also be affected. Similarly, Sgouros et al. [12] evaluated the angle formed by the left anterior clinoid process, crista galli, and right anterior clinoid process in both controls and patients with C1M, and found that this angle was smaller in children with C1M, suggesting elongation of the anterior cranial fossa in these patients. Expanding the radiological and clinical understanding of cranial base morphology, particularly with respect to the DS, in specific populations such as patients with C1M may contribute to improved surgical orientation and safer surgical planning [13]. Although previous computed tomography (CT)-based studies have evaluated various skull base and parasellar structures in patients with C1M [11,12,13], comparative CT-based investigations specifically focused on DS morphology and pneumatization remain limited. Because previous studies have suggested that morphometric alterations in C1M may extend beyond the posterior cranial fossa and involve broader regions of the skull base [11,12], we hypothesized that patients with C1M would demonstrate measurable differences in DS size and pneumatization characteristics compared with controls. Therefore, the present study aimed to evaluate DS morphology in patients with C1M and compare the findings with those of controls.

## 2. Materials and Methods

### 2.1. Ethics Statement

This retrospective CT-based investigation involving adult participants was conducted following approval from the Institutional Ethics Committee. Ethical clearance was granted on 10 October 2025, under approval number TABED 2-25-1268.

### 2.2. Study Design

The study cohort was retrospectively categorized into two groups: a control group and a C1M group. Group allocation was based on a review of hospital records and archived cranial CT examinations. Patients with C1M were evaluated between September 2019 and April 2025, whereas control subjects were selected from adult cranial CT examinations obtained between September 2024 and April 2025. The collected data included admission and discharge information, CT and/or magnetic resonance imaging (MRI) results, diagnostic assessments, treatment-related records, demographic characteristics such as age and sex, and presenting symptoms, including headache and neck pain.

### 2.3. Sample Size Determination

The number of eligible adult patients with C1M who had available cranial CT scans at our institution was limited. Accordingly, all cases fulfilling the study criteria were included, yielding a total of 60 adult patients (30 males and 30 females). A control group of 60 adults was subsequently established, with equal sex distribution between the groups. After completion of the dataset, a post hoc power analysis was performed using G*Power software version 3.1.9.7 (Heinrich Heine University, Düsseldorf, Germany) at a two-sided significance level of 0.05. For continuous morphometric variables, effect sizes were expressed as rank-biserial correlation coefficients. For DS pneumatization, the effect size was calculated as Cohen’s h = 0.56, corresponding to an achieved power of 86.3%. Overall, the final sample of 120 adults was considered adequate for the main comparative analyses.

### 2.4. Inclusion and Exclusion Criteria for the C1M Group

Adult patients with C1M were included if they had no history of meningomyelocele, had undergone surgery for C1M, and had high-quality preoperative cranial CT images available for analysis. Surgical decision-making was based on both clinical and radiological findings, including complaints such as neck pain, severe headache, balance impairment, and symptoms aggravated by Valsalva maneuvers, as well as MRI evidence of cerebellar tonsillar descent of ≥5 mm below the foramen magnum accompanied by disturbed cerebrospinal fluid flow at this level. To ensure balanced group composition, 30 adult female and 30 adult male patients with C1M were enrolled. Exclusion criteria comprised the presence of cranial base fracture or any additional cranial base pathology on preoperative CT, poor-quality CT images precluding accurate evaluation, age under 18 years, and Chiari malformation types other than C1M.

### 2.5. Inclusion and Exclusion Criteria for the Control Group

Control subjects were recruited from adult individuals who had undergone cranial CT imaging during hospital admission for various non-specific clinical reasons, including headache, trauma, traffic-related injury, or falls, and who were subsequently discharged without undergoing cranial surgery or receiving medical treatment known to affect bone metabolism. To be eligible for inclusion, subjects were required to have high-quality cranial CT images suitable for morphometric evaluation and no radiological evidence of cranial pathology. From this eligible population, 60 adults were selected for the control group, with balanced sex distribution (30 females and 30 males). Subjects were excluded if they were younger than 18 years of age, if CT image quality was insufficient for reliable analysis, or if imaging demonstrated any cranial lesion, mass, fracture, or other abnormality involving the cranial bones. Additional exclusion criteria included a history of cranial surgery, previous use of medications that could influence bone morphology, such as corticosteroids, bone-related disorders including tumors, and congenital or systemic conditions with potential effects on cranial bone structure.

### 2.6. Study Population

**Figure 1:**
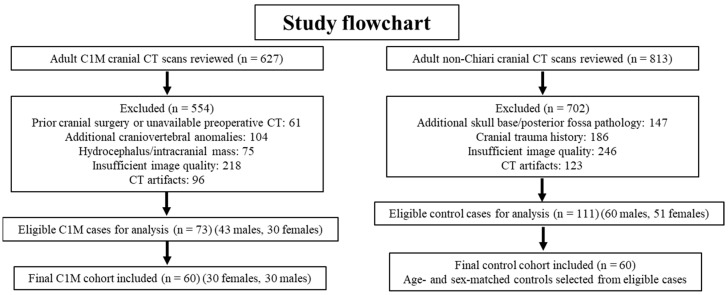
Study flowchart illustrating patient selection and exclusion processes for the C1M and control groups. C1M: Chiari malformation type I; CT: computed tomography. Control subjects were age- and sex-matched to the C1M group. Eligible cases represent patients meeting the predefined inclusion criteria after exclusion of cases with inadequate imaging quality or confounding cranial pathologies.

The study cohort consisted of 60 adults with C1M and 60 control subjects (Figure 1). The mean age was 55.25 ± 12.07 years (range, 39–84 years) in the C1M group and 55.28 ± 11.93 years (range, 30–76 years) in the control group. Both groups were balanced with respect to sex, with 30 females and 30 males in each group. Age distribution was comparable between the groups, with no significant difference observed (*p* = 0.988).

### 2.7. Computed Tomography Protocol

Cranial CT examinations were performed using a 64-detector multidetector CT scanner (VCT XTe LightSpeed; GE Healthcare, Milwaukee, WI, USA). The imaging protocol included thin-section acquisition with a slice thickness of 0.625 mm, tube voltage settings of 80 or 120 peak kilovoltage (kVp), tube current–time values between 150 and 250 milliampere-second values (mAs), a 512 × 512 matrix, and a field of view ranging from 150 to 250 mm. Images were initially obtained in the axial plane, and multiplanar reformatted coronal and sagittal images, as well as three-dimensional reconstructions, were subsequently generated. All image data were transferred to the picture archiving and communication system (PACS) and reviewed on the Advantage Workstation platform (GE Healthcare, Milwaukee, WI, USA). Both two-dimensional and three-dimensional reconstructions were used during the evaluation process. Before image analysis, multiplanar reformatted CT images were reviewed on standardized axial, coronal, and sagittal planes. Because CT examinations were routinely acquired in the supine position, image orientation was adjusted on multiplanar reformatted images when necessary to correct for head tilt and rotation, using midline anatomical structures to achieve proper alignment. The midsagittal plane was selected according to the nasal bone and adjusted using adjacent midline anatomical landmarks when necessary. All morphometric measurements and DS pneumatization assessments were performed on thin-section (0.625 mm) CT datasets using bone-window settings. The thin-section acquisition allowed high-resolution multiplanar reformations in both coronal and sagittal planes, and all measurements were verified in all three orthogonal planes to ensure measurement accuracy and reproducibility.

### 2.8. Measured Parameters

**Figure 2:**
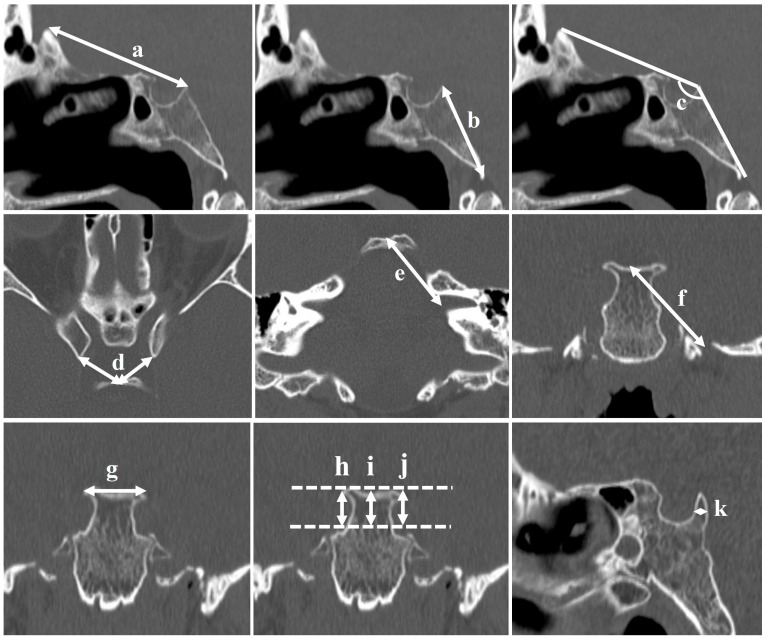
Measured parameters on standardized multiplanar reformatted CT images obtained using calibrated PACS measurement tools: a: DS-CG; b: DS-FM; c: CG-DS-FM; d: DS-ACP; e: DS-IAM; f: DS-FO; g: rPCP-lPCP; h: RLM-H; i: ML-H; j: LLM-H; k: ML-T.

A total of 11 morphometric parameters were assessed to characterize the morphology of DS (Figure 2). All measurements were performed on standardized multiplanar reformatted CT images aligned according to anatomical reference planes available in the PACS. In addition, calibrated digital measurement tools integrated into the PACS platform were used for all morphometric evaluations. These parameters were defined as follows:

- DS-CG: the linear distance between the upper corner of the crista galli and the midpoint of the superior margin of DS, measured on midsagittal reformatted CT images.
- DS-FM: the linear distance from the basion (i.e., the midpoint of the anterior edge of foramen magnum) to the superior margin of DS, measured on midsagittal reformatted CT images.
- DS-ACP: the distance extending from the midpoint of the superior margin of DS to the tip of the anterior clinoid process, assessed on axial reformatted CT images.
- DS-IAM: the distance between the midpoint of the superior margin of DS and the center of the internal acoustic meatus, measured on coronal reformatted CT images.
- DS-FO: the distance from the midpoint of the superior margin of DS to the center of the foramen ovale, determined on coronal reformatted CT images.
- CG-DS-FM: the angle formed at the midpoint of the superior margin of DS by two lines directed toward the superior aspect of the crista galli and the basion, measured on midsagittal reformatted CT images.
- rPCP-lPCP: the transverse width of the superior margin of DS, defined as the distance between the lateralmost tips of the right and left PCPs, measured on coronal reformatted CT images.
- ML-H: the vertical height of DS at the midline, measured in the superoinferior direction on coronal reformatted CT images.
- RLM-H: the superoinferior height at the right lateral aspect of DS, defined as the distance from the highest point of the right PCP to the base of DS, measured on coronal reformatted CT images.
- LLM-H: the superoinferior height at the left lateral aspect of DS, defined as the distance from the highest point of the left PCP to the base of DS, measured on coronal reformatted CT images.
- ML-T: the midline thickness of DS, corresponding to its anteroposterior diameter, measured on midsagittal reformatted CT images.

### 2.9. Evaluation of Dorsum Sellae Pneumatization

**Figure 3:**
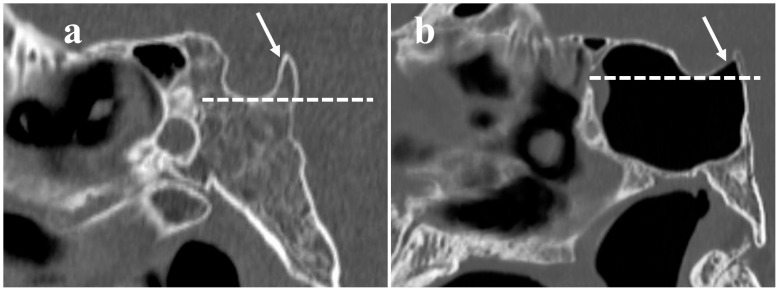
Dorsum sellae pneumatization: (**a**) absence; (**b**) presence. Arrows indicate the region of interest, and the dashed line indicates the reference line used for assessment of dorsum sellae pneumatization.

Presence or absence of radiologically defined DS pneumatization was evaluated using midsagittal CT scans (Figure 3), according to previously described CT-based criteria [10]. For the assessment of pneumatization, a line passing through the base of DS was used as a reference, and any air-containing area located above this line was considered as DS pneumatization.

### 2.10. Statistical Analysis

Statistical analyses were performed using IBM SPSS Statistics version 25.0 software (IBM Corp., Armonk, NY, USA). Measurements were independently performed by two radiologists experienced in cranial CT anatomy, and the final quantitative values used for analysis were calculated as the mean of the two measurements. Interobserver reliability was assessed using intraclass correlation coefficients (ICCs). Continuous variables were expressed as median (Q1–Q3), and comparisons between the control and C1M groups were performed using the Mann–Whitney U test. For bilaterally located parameters, namely DS-ACP, DS-IAM, and DS-FO, right- and left-sided measurements were obtained separately. As no significant side-to-side difference was identified, the mean of the right and left measurements was calculated for each subject and used for between-group comparisons. The presence or absence of pneumatization was determined by consensus between the two investigators. Categorical variables were presented as number (percentage), and the presence of DS pneumatization was compared between groups using Fisher’s exact test because of the low expected cell counts. To account for the potential inflation of type I error due to multiple comparisons, a Benjamini–Hochberg false discovery rate (FDR) correction was additionally applied to the *p* values obtained from the primary group comparisons. All tests were two-tailed, and a *p* value < 0.05 was considered statistically significant.

## 3. Results

### 3.1. Analysis of the Repeatability of Measurements

Interobserver reliability was high, with ICC values ranging from 0.940 to 0.990 (all *p* < 0.001).

### 3.2. Relationship of the Dorsum Sellae with Surrounding Structures

The spatial relationship between DS and adjacent skull base structures was compared between the control and C1M groups (Table 1). No significant difference was observed in the DS-CG measurement between the control group (54.15 mm) and the C1M group (52.30 mm) (*p* = 0.887). In contrast, the DS-FM distance was significantly shorter in the C1M group than in the control group (43.15 mm vs. 46.00 mm, respectively; *p* < 0.001). The DS-ACP distance was significantly greater in the C1M group compared with the control group (17.20 mm vs. 15.10 mm, respectively; *p* < 0.001). No statistically significant difference was found in the DS-IAM measurement between the control and C1M groups (40.30 mm vs. 39.35 mm, respectively; *p* = 0.086). Likewise, the DS-FO distance was comparable between the two groups (35.30 mm vs. 36.25 mm, respectively; *p* = 0.869). However, the CG-DS-FM angle was significantly greater in the C1M group than in the control group (133.44° vs. 125.30°, respectively; *p* < 0.001). Overall, these findings suggest that C1M is associated with significant alterations in selected parameters describing the relationship of the DS with surrounding skull base structures.

### 3.3. Direct Morphometric Parameters of the Dorsum Sellae

The direct morphometric parameters of the DS were compared between the control and C1M groups (Table 1). The rPCP-lPCP distance was significantly lower in the C1M group than in the control group (20.00 mm vs. 22.00 mm, respectively; *p* = 0.003). The ML-H value was significantly greater in the C1M group compared with the control group (5.04 mm vs. 3.48 mm, respectively; *p* < 0.001). Similarly, the RLM-H measurement was significantly higher in the C1M group than in the control group (4.63 mm vs. 3.04 mm, respectively; *p* < 0.001). The LLM-H value was also significantly greater in the C1M group (4.80 mm vs. 3.73 mm, respectively; *p* < 0.001). In addition, the ML-T measurement was significantly higher in the C1M group than in the control group (2.25 mm vs. 1.54 mm, respectively; *p* < 0.001). Overall, these findings indicate that individuals with C1M exhibit significant differences in several direct morphometric dimensions of the DS, characterized by reduced transverse width and increased vertical and anteroposterior dimensions.

### 3.4. Pneumatization of the Dorsum Sellae

The presence of DS pneumatization was compared between the control and C1M groups. DS pneumatization was identified in 3 of 60 subjects (5.0%) in the control group and in 14 of 60 subjects (23.3%) in the C1M group. This difference was statistically significant (*p* = 0.007, Cohen’s h = 0.557). Accordingly, DS pneumatization was significantly more frequent in individuals with C1M than in controls, with an odds ratio of 5.783 (95% CI: 1.566–21.347).

### 3.5. False Discovery Rate Analysis

To account for the potential inflation of type I error due to multiple comparisons, a Benjamini–Hochberg FDR correction was applied to all primary group comparisons. Following FDR adjustment, all findings that were statistically significant in the primary analyses remained statistically significant, whereas non-significant findings remained non-significant. The corresponding FDR-adjusted *p* values were as follows: DS-CG, *p* = 0.968; DS-FM, *p* < 0.001; DS-ACP, *p* < 0.001; DS-IAM, *p* = 0.103; DS-FO, *p* = 0.948; CG-DS-FM, *p* < 0.001; rPCP–lPCP, *p* = 0.005; ML-H, *p* < 0.001; RLM-H, *p* < 0.001; LLM-H, *p* < 0.001; ML-T, *p* < 0.001; and DS pneumatization, *p* = 0.010.

## 4. Discussion

C1M is a complex craniovertebral anomaly reported with a prevalence ranging from 0.24% to 3.6% [14]. It is classically defined by caudal displacement of the cerebellar tonsils by at least 5 mm below the level of the foramen magnum [14]. Although its precise pathophysiological basis remains incompletely understood, aberrant development of the occipital somites derived from the paraxial mesoderm is considered a central etiological mechanism [15]. C1M has traditionally been regarded as a disorder primarily involving the posterior cranial fossa, where an approximately 25% reduction in volume has been described [15,16]. This volumetric restriction may result in overcrowding of the hindbrain and contribute to a broad clinical spectrum, including headache, facial sensory disturbances, and pain [15,16,17]. While the osseous abnormalities of C1M predominantly affect the posterior fossa, accumulating evidence suggests that skeletal variations are not confined to this region and may extend to other compartments of the cranial base [11,12]. Compared with controls, patients with C1M have been reported to exhibit a longer anterior cranial fossa, increased sphenoid sinus volume, reduced sellar volume, and a shallower middle cranial fossa. These observations reflect a growing interest in defining cranial base alterations beyond the posterior fossa in C1M [11,12,18,19]. Nevertheless, the morphology of the DS in this patient group remains insufficiently investigated. In light of the expanding anatomical perspective on the cranial base in C1M, a focused evaluation of DS size and spatial relationships may offer additional insight into the structural phenotype of this malformation.

Data regarding the spatial relationships of the DS with surrounding skull base structures in patients with C1M remain limited in the literature [12,13], although such information may be important for a more comprehensive understanding of skull base anatomy in this condition. Sgouros et al. [12] analyzed preoperative MRI findings in 30 children with symptomatic isolated C1M, including 14 without syringomyelia and 16 with syringomyelia, and compared them with 42 age-matched controls. They found that the CG-DS-FM angle was greater in both C1M subgroups than in controls, measuring 145° in patients without syringomyelia and 151° in those with syringomyelia, compared with 135° in the control group (*p* < 0.001). They also reported that the DS-FM distance, representing posterior fossa depth, was 50 mm in the C1M group without syringomyelia, 40 mm in the C1M group with syringomyelia, and 46 mm in controls; within the C1M cohort, this distance was significantly smaller in patients with syringomyelia (*p* = 0.009) [12]. Alpergin et al. [13] evaluated PCP morphology in 52 patients with C1M and 71 controls. Their findings showed that the distances from the PCP to the crista galli and the foramen magnum, reflecting anterior fossa length and posterior fossa depth, respectively, were altered in the C1M group, suggesting a longer anterior cranial fossa and a relatively shallow posterior fossa. Moreover, measurements between the PCP and the superior orbital fissure, foramen rotundum, and foramen ovale, which were used to characterize middle cranial fossa width, indicated that the middle fossa was narrower in patients with C1M than in controls [13]. In line with these observations, the increased CG-DS-FM angle and reduced DS-FM distance in the present study indicate altered skull base angulation at the level of the DS together with relative shallowing of the posterior fossa in C1M.

The literature contains limited data regarding the size of the DS in patients with C1M [20,21], although such information may contribute to a more comprehensive understanding of skull base anatomy in this condition. Patel et al. [20] initially observed apparently enlarged pituitary glands on MRI in several patients with Chiari malformation type II (C2M). This incidental finding subsequently prompted them to conduct a systematic MRI-based evaluation in a cohort of 21 patients. Their analysis demonstrated that, compared with controls, patients with C2M exhibited a taller pituitary gland in the absence of intrinsic pathology, a longer tuberculum sellae, a shorter DS, and a shallower sella turcica. The authors suggested that the shallow sella turcica may lead to misinterpretation of the pituitary gland as enlarged on MRI, since a normal gland may appear relatively taller within this altered sellar configuration [20]. Although C2M has a distinct developmental and pathophysiological background compared with C1M, these observations were considered relevant because they suggested that Chiari-related cranial base abnormalities may also involve DS morphology. Based on the above study conducted in patients with C2M, Dolgun et al. [21] evaluated the same sellar and pituitary morphometric relationship in adult patients with C1M. In their MRI-based study including 50 patients with C1M and 50 controls, pituitary gland height was statistically similar between the groups, whereas both tuberculum sellae height and DS height were significantly lower in the C1M group than in controls. These findings suggest that a relatively shallow sella may create the false impression of pituitary enlargement on MRI despite the absence of intrinsic pituitary pathology [21]. In our CT-based analysis, patients with C1M also showed significant alterations in DS morphology, characterized by a reduced rPCP–lPCP distance and increased ML-H, RLM-H, LLM-H, and ML-T measurements compared with controls. Although these findings differ from some previous MRI-based observations, such discrepancies may be related to differences in imaging modality, morphometric methodology, and the specific DS-related parameters evaluated across studies.

Information on DS pneumatization remains scarce in the literature, and, to the best of our knowledge, no previous study has specifically evaluated its presence in patients with C1M. In a CT-based study of 1080 otherwise normal subjects aged 1–90 years, Alpergin et al. [22] reported DS pneumatization in 32.8% overall, with a prevalence of 21.2% in children and 36.1% in adults. Likewise, Atadağ et al. [10] examined a pediatric sample of 360 children aged 1–18 years and found DS pneumatization in 19.7%, again indicating that its pneumatization increases with age. However, neither of these studies focused on C1M. In the Chiari literature, Alpergin et al. [13] evaluated PCP rather than DS in a CT series including 52 patients with C1M and 71 controls, and demonstrated that PCP pneumatization was significantly more frequent in the C1M group than in controls (38.5% vs. 19.7%, *p* < 0.001). Consistent with the concept that osseous aeration around the sellar region may be altered in C1M, our findings showed that DS pneumatization was also significantly more common in the C1M group than in controls (23.3% vs. 5.0%, *p* = 0.007; OR, 5.783; 95% CI, 1.566–21.347). Moreover, a separate 3D CT study demonstrated that patients with C1M had a 38% greater sphenoid sinus volume and a 27% smaller sella turcica area compared with controls [11]. These findings suggest that the higher rate of DS pneumatization observed in our C1M group may be related to increased sphenoid sinus aeration and concomitant morphologic alterations involving the sellar region.

The pathogenesis of C1M remains controversial, and several competing theories regarding its developmental and morphometric basis have been proposed in the literature. Goel et al. [23,24] suggested that at least some craniovertebral alterations associated with C1M may represent adaptive or protective responses to underlying craniovertebral instability rather than isolated congenital malformations, even proposing the term “Chiari formation” instead of “Chiari malformation” in this context. In parallel with these conceptual discussions, increasing attention has been directed toward skull base morphometry in C1M. In a comprehensive review of morphometric studies, Shuman et al. [25] emphasized that although reduced clivus length, posterior fossa abnormalities, and other cranial base measurements (e.g., McRae line length, basal angle, and supraocciput length) have frequently been associated with C1M, no single morphometric parameter has consistently explained the anatomical and clinical heterogeneity of the disease. The authors further suggested that additional morphometric markers beyond tonsillar descent alone may improve anatomical characterization of C1M [25]. Nwotchouang et al. [11] identified significant CT-based clival and sphenoid sinus dysmorphism in adult patients with C1M using three-dimensional morphometric analysis. Sgouros et al. [12] demonstrated abnormal geometrical measurements involving the entire skull base, including DS-related parameters, in children with C1M. These observations support the concept that morphometric alterations associated with C1M may extend beyond the posterior cranial fossa and involve broader regions of the skull base. Previous studies have suggested that paraxial mesodermal insufficiency and consequent underdevelopment of the basichondrocranium may contribute to abnormal cranial base growth in C1M [12,26,27]. Because the DS develops from the basisphenoid portion of the central skull base, developmental alterations affecting adjacent spheno-clival structures may theoretically also influence DS morphology. Although the precise mechanisms underlying these DS-related alterations remain unclear, the observed morphometric pattern may reflect broader morphologic differences of the spheno-clival region and central skull base in C1M. Within this evolving morphometric framework, the present study specifically focused on DS and demonstrated significant differences in several DS-related dimensions and pneumatization characteristics between patients with C1M and controls. Although the retrospective case–control design of the present study does not permit causal inference, the observed DS-related differences suggest that DS morphology may represent another skull base parameter associated with C1M. Our findings may also be compatible with previously proposed hypotheses regarding cranial base morphological alterations in C1M.

This study, which compared adult subjects with C1M and controls, should be interpreted in light of several limitations. First, the sample size was moderate, and larger cohorts may provide a more robust characterization of DS morphology in both groups. Second, the measurements were obtained from two-dimensional CT images; therefore, future studies using three-dimensional CT-based analyses may yield a more detailed evaluation of DS anatomy. Third, the control group consisted of adults who underwent cranial CT for various clinical indications, including trauma, headache, traffic accidents, and falls, rather than asymptomatic healthy volunteers. Therefore, a degree of selection bias cannot be excluded. However, truly healthy asymptomatic adults are generally unavailable in retrospective hospital-based CT archives. To minimize potential bias, all control subjects were carefully screened to exclude cranial pathology, prior cranial surgery, and conditions potentially affecting cranial bone morphology. Accordingly, these individuals were considered suitable for comparative morphometric evaluation within the context of the present retrospective study. Fourth, because the present study primarily focused on direct morphometric evaluation of the DS, the relationship between DS morphology and overall cranial dimensions was not specifically analyzed. Future retrospective or prospective studies examining additional cranial morphometric parameters, including skull width, skull height, cranial base dimensions, and volumetric measurements, may contribute to a more comprehensive understanding of the anatomical characteristics of DS in C1M. Fifth, DS pneumatization was defined according to previously published CT-based criteria and was assessed on midsagittal CT images [10,22]. Although this approach has been adopted in prior radiological studies, it represents an imaging-based operational definition of DS pneumatization. Future investigations incorporating multiplanar and three-dimensional assessments may further refine the radiological characterization of DS pneumatization and improve understanding of its prevalence and morphological characteristics in patients with C1M. Sixth, DS pneumatization was evaluated using a binary present/absent classification rather than a graded or volumetric assessment. Therefore, the extent and anatomical variation of pneumatization could not be analyzed in detail. Future CT-based studies incorporating three-dimensional volumetric or classification-based analyses may provide a more comprehensive characterization of DS pneumatization patterns in C1M. Seventh, the present cohort consisted of adult patients with clinically and radiologically confirmed C1M who had undergone surgical evaluation and treatment. Future studies including incidental or minimally symptomatic cases may contribute to a more comprehensive understanding of skull base anatomy across the broader clinical spectrum of C1M. Eighth, although interobserver reliability was evaluated, intraobserver repeatability was not separately assessed. In addition, measurements were not performed under formal blinding to group allocation. These factors may represent potential methodological limitations affecting measurement reproducibility. Future morphometric investigations incorporating intraobserver reliability analyses and blinded measurement protocols may further contribute to the validation and reproducibility of DS-related measurements. Ninth, although the groups were age-matched overall, age-adjusted analyses for DS pneumatization were not specifically performed despite previous reports demonstrating age-related increases in DS pneumatization. Future studies using multivariable statistical models may further clarify the independent relationship between C1M and DS pneumatization. Despite these limitations, our findings may still provide useful baseline data for a more comprehensive understanding of DS morphology and may serve as a basis for future investigations.

## 5. Conclusions

Patients with C1M exhibit significant differences in both the spatial relationships and morphometric characteristics of the DS compared with controls. The increased CG-DS-FM angle and reduced DS-FM distance indicate altered skull base angulation at the level of the DS together with relative shallowing of the posterior fossa in C1M. In addition, DS pneumatization was more frequent in the C1M group. CT-based evaluation of the DS may provide useful anatomical information and contribute to a better understanding of skull base configuration and sellar imaging findings in patients with C1M.

## Figures and Tables

**Table 1:** Comparison of dorsum sellae-related morphometric parameters between the control and C1M groups.

Parameter	Control Group Median (Q1–Q3)	C1M Group Median (Q1–Q3)	*p*-Value	Rank-Biserial Correlation
DS-CG	54.15 (47.88–55.85)	52.30 (50.12–55.12)	0.887	−0.015
DS-FM	46.00 (43.45–49.72)	43.15 (41.20–45.68)	<0.001	−0.391
DS-ACP	15.10 (14.17–16.90)	17.20 (15.08–26.30)	<0.001	0.391
DS-IAM	40.30 (39.05–41.02)	39.35 (36.55–41.48)	0.086	−0.182
DS-FO	35.30 (34.40–37.35)	36.25 (33.45–38.42)	0.869	0.018
CG-DS-FM	125.30° (118.14–129.18)	133.44° (126.13–140.53)	<0.001	0.485
rPCP–lPCP	22.00 (18.90–23.90)	20.00 (18.70–21.60)	0.003	−0.314
ML-H	3.48 (2.62–4.32)	5.04 (4.53–5.40)	<0.001	0.597
RLM-H	3.04 (2.94–3.20)	4.63 (4.30–5.44)	<0.001	0.906
LLM-H	3.73 (3.53–4.29)	4.80 (4.52–5.11)	<0.001	0.732
ML-T	1.54 (1.34–2.07)	2.25 (1.92–2.85)	<0.001	0.653

Data are presented as median (Q1–Q3). Between-group comparisons were performed using the Mann–Whitney U test. Effect sizes are presented as rank-biserial correlation coefficients. Positive values indicate higher measurements in the C1M group relative to controls, whereas negative values indicate lower measurements. Abbreviations: DS, dorsum sellae; CG, crista galli; FM, foramen magnum; ACP, anterior clinoid process; IAM, internal acoustic meatus; FO, foramen ovale; PCP, posterior clinoid process; ML-H, midline height; RLM-H, right lateral morphometric height; LLM-H, left lateral morphometric height; ML-T, midline thickness.

## Data Availability

Available with the corresponding author (Burak Bahadır, e-mail: burakbahadirmd@gmail.com) on request.

## Abbreviations

DS	dorsum sellae
CT	computed tomography
C1M	Chiari malformation type I
PCP	posterior clinoid process
PACS	the picture archiving and communication system
mAs	milliampere-second values
kVp	peak kilovoltage
ICC	intra-class correlation coefficients
C2M	Chiari malformation type II
